# The impact of psychiatric disorders of parents on the severity of substance use disorder in their offspring

**DOI:** 10.1192/j.eurpsy.2022.2119

**Published:** 2022-09-01

**Authors:** S. El-Makawi, S. Abou El Magd, N. Nagy

**Affiliations:** Cairo University, Psychiatry, Cairo, Egypt

**Keywords:** parent mental disorder, substance use, addiction severity index

## Abstract

**Introduction:**

There is a lack of evidence in the literature about the impact of offspring addiction and their parents’ mental health. Objective is to explore psychiatric disorders in parents of patients with substance dependence and their effect on the severity of their addiction.

**Objectives:**

To evaluate psychiatric disorders in parents of patients with substance dependence and their effect on the severity of their addiction.

**Methods:**

This is a cross sectional study contained group (A) 150 patients diagnosed with substance dependence according to DSM-IV. Patients were recruited form Psychiatry and Addiction Hospital of Cairo University. Group (B) included one or both parents of the patients group. The Addiction Severity Index (ASI) was used for the patients.

**Results:**

In the patients group, mean age was 24.89 (±4.52). 96% of them were males 4% were females. In the parent group, mean age was 51.59 (±5.48). 84% of them were mothers 16 % were females. Patients group was associated with moderate degree of education (46.7%), 73.3% were single and 64% were unemployed. Anxiety disorders (80%) and depressive disorders (69.3%) were the most prevalent among parents group. ASI score was statistically significant in predicting the incidence of parents psychotic and schizoid disorders (100% sensitivity, 86.3% specificity, AUC=0.887, P value <0.0001).Besides we found a significant correlation between patients ASI scores and parents psychiatric disorder.

**Conclusions:**

This result emphasizes the relation between patient substance use disorder and psychiatric disorder of their parents. Patients ASI score may be a possible measure for parents mental disorder. Further research is needed to validate our findings.

**Disclosure:**

No significant relationships.

